# Special Issue: Emergency Medicine: Clinical Advances and Challenges in Diagnosis and Treatment, 2nd Edition

**DOI:** 10.3390/jpm15100493

**Published:** 2025-10-14

**Authors:** Carmen Gabriela Williams, Daian-Ionel Popa, Ovidiu Alexandru Mederle

**Affiliations:** 1 Emergency Department, Emergency Clinical Municipal Hospital, Gheorghe Dima Street Number 5, 300079 Timisoara, Romania; williams.carmen@umft.ro (C.G.W.);; 2 Doctoral School, Faculty of General Medicine, Victor Babes University of Medicine and Pharmacy, 300041 Timisoara, Romania

## 1. Introduction

The development of emergency medicine brings both challenges and opportunities. With the joint efforts and dedication of healthcare professionals, alongside the integration of continuously updated medical technologies and concepts, the field is steadily moving toward historic advancements. Emergency medicine is a dynamic specialty, constantly evolving to enhance patient outcomes and ensure that safety remains the highest priority during critical situations.

This Special Issue, “Emergency Medicine: Clinical Advances and Challenges in Diagnosis and Treatment, 2nd Edition”, has brought together thirteen high-quality contributions, including nine original research articles, three reviews, and one case report.

## 2. An Overview of Published Articles

Marza et al. (Contribution 1) describe a rare and dramatic complication of synchronized cardioversion for unstable supraventricular tachycardia: complete heart block with recurrent cardiac arrests, ultimately requiring permanent pacemaker implantation. The case is striking for its detailed chronology, systematic troubleshooting, and eventual success in repositioning transcutaneous pacing electrodes. Unexpected deterioration remains a reality in resuscitation practice. Case reports within this Special Issue remind us that even routine interventions such as synchronized cardioversion may precipitate bradyarrhythmic arrest. Similar observations have been documented in larger studies, where electrical cardioversion occasionally triggers sinus node dysfunction or conduction disturbances requiring pacing support [1]. The 2021 European Resuscitation Council Guidelines reaffirm the need for vigilance in rhythm-specific therapy, rapid recognition of arrest, and preparation for advanced pacing or pharmacological escalation in case of refractory bradyarrhythmia [2].

Guan et al. (Contribution 2) present a timely and innovative study addressing a common clinical dilemma: whether residents of residential aged care facilities (RACFs) who contribute to the emergency department (ED) after a fall can be safely discharged. The authors developed and internally validated two models, culminating in the simplified four-variable Discharge Eligibility after Fall in Elderly Residents (DEFER) score. Older adults represent one of the fastest-growing patient cohorts in the ED. Predicting safe discharge after a fall, particularly for residents of aged care facilities, is a recurrent challenge—models such as the DEFER score attempt to balance clinical risk with respect for patient preferences. Broader evidence confirms that falls are multifactorial, influenced by polypharmacy, frailty, and cognitive impairment [3]. However, existing screening tools have shown limited reproducibility in ED settings [4]. International experts argue that emergency care for older adults must be redesigned to integrate risk stratification, advance care planning, and system-level adaptations [5].

Hsieh et al. (Contribution 3) contribute significantly and innovatively to infectious disease emergency care by evaluating and modifying existing scoring systems for patients with Vibrio bacteremia. The study demonstrates that the Mortality in Emergency Department Sepsis (MEDS) score, when modified with simple laboratory markers such as blood urea nitrogen (BUN) and arterial pH, provides superior predictive accuracy compared with traditional models. Vibrio vulnificus sepsis, though uncommon, is the most fulminant and deadly infectious emergency presenting in medicine, with a greater than 50% mortality rate even when treated appropriately with antibiotics and intensive care support. Therefore, updating current tools of sepsis risk stratification, applicable more by adding readily available biochemical parameters, is another move toward making an early clinical decision better and improving survival outcomes in this high-risk population [6].

Acute chest pain remains a frequent and diagnostically challenging reason for visits to the emergency department, requiring rapid exclusion of benign causes while promptly identifying acute coronary syndromes (ACSs). In this setting, the article by Piccioni (Contribution 4) and colleagues provides valuable insights by assessing a multi-marker strategy that combines high-sensitivity troponin I (hsTnI) with two emerging biomarkers, soluble ST2 (sST2) and soluble urokinase plasminogen activator receptor (suPAR). Given that acute chest pain represents one of the most frequent and diagnostically demanding presentations in the emergency department—requiring clinicians to swiftly discriminate between benign causes and high-risk entities such as acute coronary syndromes (ACSs)—the work of Eggers et al. offers compelling evidence that combining high-sensitivity troponin T (hsTnT) with additional biomarkers like copeptin can significantly improve early diagnostic accuracy and expedite safe clinical decision-making in suspected ACS patients [7].

Buleu et al. (Contribution 5) offer a valuable and timely contribution to acute stroke management. By examining the performance of emergency departments in relation to door-to-needle time across day and night shifts, the authors provide important insights into operational challenges and opportunities for improvement. Stroke care remains time-critical. Contributions addressing code-stroke performance, mechanical thrombectomy via alternative vascular routes, and misdiagnosis risk highlight procedural and diagnostic personalization. The European Stroke Organisation guidelines emphasize individualization of thrombolysis and thrombectomy strategies, depending on comorbidities and vascular anatomy [8,9].

The review article by Moroi et al. (Contribution 6) provides an in-depth and timely examination of one of the most challenging emergencies in maternal care: venous thromboembolism during pregnancy. The authors successfully present a balanced and comprehensive overview of epidemiology, risk factors, diagnostic difficulties, and therapeutic strategies. Thromboembolism of the venous system in pregnancy continues to be one of the major killers of women worldwide. It poses a unique diagnostic and therapeutic challenge because, on one hand, there are overlapping physiological changes, and on the other hand, both mother and fetus need protection. In this regard, Bates et al. have come up with an authoritative practice synthesis that outlines evidence-based strategies for clinical assessment, imaging selection, and choice of anticoagulation protocols at different stages of pregnancy. This emergency and obstetric team will work in a high-risk situation needing fast decisions based on guidelines [10].

Kim et al. (Contribution 7) provide a comprehensive and timely evaluation of mechanical CPR use in the management of out-of-hospital cardiac arrest, with a particular focus on the COVID-19 pandemic period. By leveraging a robust, nationwide dataset, the authors deliver necessary evidence on survival, neurological outcomes, and return of spontaneous circulation, while carefully contrasting outcomes between manual and device-assisted resuscitation. Mechanical cardiopulmonary resuscitation has been purposely brought into the debate in cases of out-of-hospital cardiac arrest because it is often blamed for poor neurological outcomes and overall survival, when indeed a valuable contribution, such as that made by Couper et al. [11], through an analysis of randomized trials together with observational cohorts comparing device-assisted resuscitation to conventional manual techniques, proves otherwise. While it may not uniformly improve outcomes when used routinely, mechanical CPR can offer significant operational advantages during prolonged resuscitation efforts and transport and in high-risk infectious settings, wherein provider safety and resource allocation are critical determinants of care quality [11].

The research from Hahnenkamp (Contribution 8) present an exemplary innovation model in rural emergency care, offering a well-structured evaluation of the RuralRescue initiative in northeastern Germany. The authors successfully demonstrate how integrating tele-emergency physician supervision, geolocation-based responder apps, and structured data-driven triage can significantly improve timeliness and quality. Rural emergency care continues to suffer from problems of delayed response time, lack of availability of specialists, and uncoordinated resources. In such a scenario, the studies by Hong et al. and Zakariah et al. would be most relevant to prove that telemedicine-supported physician oversight integrated with digitally coordinated first responders significantly improves prehospital care delivery in underserved regions. Remote supervision combined with geolocation-based alert systems and structured triage protocols presents measurable improvements in clinical efficiency as well as patient outcomes and is scalable on different healthcare infrastructures [12,13].

Iancu’s work (Contribution 9) address a rare but clinically critical topic: mechanical thrombectomy via the transbrachial approach for acute ischemic stroke in patients with aortic pathologies. The authors enrich the literature by combining a carefully described case from their practice with an extensive review of nine published cases, thereby filling a notable gap in evidence. Other access routes for mechanical thrombectomy are seldom needed but become very important in patients with prohibitive aortic or peripheral vascular anatomy; thus, the case series presented by Iancu et al. provides valuable insight by documenting successful transbrachial and transradial approaches when standard transfemoral access could not be achieved. This is an excellent demonstration that procedural flexibility is also very much involved, as timely vascular reassessment and fast conversion to upper extremity access can preserve the chances of recanalization without compromising safety in these anatomically complex stroke patients [14].

The paper by Hodgson et al. (Contribution 10) pioneers the use of artificial intelligence and machine learning to optimize emergency department (ED) operations. The authors present a methodologically rigorous study, training a non-linear ML model on more than 49,000 patient encounters and prospectively implementing a personalized vertical processing pathway (VPP) over 13 weeks. Artificial intelligence and machine learning have become a transformation plan in emergency departments that will improve the precision of triage and resource allocation, enhancing operations’ overall efficiency. In this context, Fernandes et al. and Takeda et al. presented solid proof by demonstrating that predictive models based on data can effectively preempt patient disposition, admission risk, and wait time dynamics compared to assessments led by clinicians using traditional methods. Their studies have validated that frameworks for triage based on machine-learning algorithms improve patient flow as well as minimize the extent of overcrowding, personalized decision pathways that will suit varying demands in EDs [12,15].

Savioli’s study (Contribution 11) provides a significant and timely contribution to the ongoing debate on triage accuracy in emergency departments, focusing specifically on the geriatric population—one of the most vulnerable and complex groups of patients. Drawing on more than 420,000 ED admissions over six years, the authors conduct a robust real-life comparison between four-level and five-level triage systems (4LT and 5LT) and their impact on under-triage (UT), over-triage (OT), waiting times, and crowding indices. In this context, the studies by Grossmann et al. and Türkoğlu et al. provide valuable evidence that standard triage protocols may inadequately capture severity in geriatric patients, with high misclassification rates observed across four- and five-level systems. Their findings emphasize that older adults are disproportionately affected by prolonged waiting times and inappropriate resource allocation, even within structured triage frameworks, underscoring the need for age-adapted triage modifiers and continuous algorithm refinement [16,17].

Anna Ingielewicz’s work (Contribution 12) offer a thoughtful and comprehensive exploration of one of emergency medicine’s most fundamental yet elusive challenges: the pursuit of an ideal triage system. The authors carefully review and compare the most widely used five-level systems, ATS, CTAS, ESI, and MTS, while contextualizing their strengths, weaknesses, and practical limitations in real-world emergency department (ED) settings. An ideal triage system is the most elusive and hotly contested quality improvement that one can undertake in emergency medicine since even today’s five-level tools cannot escape challenges regarding their accuracy, validity, or consistency among different patient populations. It is within this context that the works of Farrohknia et al. and Zachariasse et al. are placed since they present a veritable accounting of the major triage frameworks—ATS, CTAS, ESI, and MTS—not only based on validity and reproducibility but also specific performances in high-risk subgroups such as pediatric and geriatric patients. Indeed, disparities continue to exist even when fully robust systems are implemented, especially at intermediate urgency categories where continuous refinement and contextualization, added by complementary risk modifiers, are justified [18,19].

Recognition of prehospital stroke has continued to bedevil even the most advanced emergency systems, as recent evidence corroborated by findings reported by Jalali et al. (Contribution 13) attests. There is still a diagnostic gap in the field. Contemporary studies sustain that even when structured tools—FAST or Cincinnati scale—are included, the paramedics misclassify up to one-third of the cases regarding the same patient complaining about suspected stroke, posterior circulation events, presentations with isolated dizziness, confusion, and atypical symptoms [20,21].

In conclusion, the articles presented in this Special Issue encompass a diverse array of subjects. We extend our sincere appreciation to the authors for their significant contributions. Their dedicated efforts have demonstrated their commitment to research endeavors. The insights and understanding they have contributed have a remarkable capacity to shape the direction of emergency medicine, advancing the notion of personalized healthcare. We encourage readers to delve into the articles and recognize the transformative effects of innovative interventions as they seek to enhance the standard of patient care.
